# Playing for Keeps: Advancing Serious Games in Neurology Education

**DOI:** 10.1212/NE9.0000000000200222

**Published:** 2025-06-11

**Authors:** Megan G. Jiao, Zachary N. London

**Affiliations:** 1 McGovern Medical School, University of Texas Health Science Center at Houston, Houston, TX; and; 2 Department of Neurology, University of Michigan, Ann Arbor, MI.

Game-based learning can enhance interest, engagement, and application of knowledge across educational settings, including the clinical neurosciences. Serious games, a subset of game-based learning, are broadly defined as games designed for purposes beyond entertainment, such as education.^1^ Key components of serious games include rules, interactivity, and game elements (e.g., competition, rewards, or social interaction). Players make choices within the rules, receive feedback, and modify behavior in response. This cycle, called the core gameplay loop, reinforces learning objectives, promotes knowledge retention, and motivates continued participation.^2^

Serious games have gained traction in medical education because they promote active learning and improve knowledge retention over passive methods. Their playful elements can boost learner motivation and satisfaction while offering a safe environment for learners to apply knowledge and practice techniques. When well-designed, serious games foster understanding of complex systems by embedding those systems into the gameplay itself.^3,4^

In this issue of *Neurology*®*: Education*, Heidrich et al.^5^ report a randomized study evaluating a novel interactive, video-based, digital resource (referred to as a serious game) for teaching the diagnosis and management of neurologic emergencies to medical students in Germany. Their curriculum development emphasized both medical knowledge and clinical workflows. Students using the resource retained more knowledge over time than those learning through seminars. More also reported that the experience helped them organize their thinking, understand clinical workflows, and prepare for clinical practice.

Learner feedback offered valuable insights into the process of developing this type of curricular resource. Some noted that the interactive decision pathway was time consuming, and that individualized feedback was limited, suggesting that future iterations might benefit from a moderated hybrid format.

The authors found that creating high-fidelity digital resources is time consuming, expensive, and limited by clinicians' lack of technical knowledge. One takeaway from their experience is the need to design serious games that are less resource-intensive but still effective. Notably, the process of developing the resource from scratch was highly satisfying for their team. This is a reminder that educator engagement, although often overlooked, is an important goal in education research.

The classification of resource of Heidrich et al. presents a challenge in interpreting this study in the broader context of educational resources. It is unclear whether the resource qualifies as a serious game, a simulation, or both. Simulation is a form of experiential learning that reproduces real-world situations or environments. Unlike serious games, simulations do not necessarily include goals, rules, and game mechanics.^6,7^

While the resource of Heidrich et al. includes interactive decision pathways, there are no other game elements described to distinguish it from a simulation. It is also impossible to reproduce their game based solely on their descriptions, preventing further evaluation by readers in their local teaching contexts. This does not diminish the educational value of the resource, but it may limit how easily their findings generalize to other serious game research.

Perhaps, the best way to enable readers to classify the resource would be to share it. While it is exciting to learn about successful educational interventions, educators and game developers would be better positioned to translate lessons to their own educational contexts if they could experience the resource directly. It would also allow them to confirm alignment between objectives, content, and assessments and clarify whether the mode of instruction drives the observed differences between groups. This can also facilitate developing best practices, such as standardized classification of resources.

In medical research, reproducibility is supported through transparency, detailed methodology, shared code and analysis, and independent replication. Research on serious games should be held to the same standard, either by including the resources themselves or by providing sufficient detail to replicate them. This will foster discourse and deepen understanding.

This raises a broader issue: the generalizability of research about serious games. Even under strict definitions, serious games vary widely in format (e.g., digital vs analog) and gameplay (e.g., solo, cooperative, or competitive; narrative-driven vs mechanics-driven). This heterogeneity complicates broad conclusions about their educational value.

Because serious games are a nascent field in neurology education, many medical educators lack experience in game development. This disconnect can lead to communication gaps with technical collaborators and could result in games that fail to engage or educate effectively. Just as new investigators receive training in research methods, aspiring serious game developers should seek training in game design. They may also benefit from engaging learners in the development and evaluation of serious games to leverage their existing familiarity with games and better address their learning needs.

As Heidrich et al. suggest, serious games can be rewarding for both learners and educators. Their work can serve as both a case study and an inspiration. Readers can carry forward their enthusiastic spirit to create or apply games in their own educational spaces. There is a growing evidence base for serious games in neurology education, but many studies lack sufficient evaluation of their validity, impact, sustainability, and generalizability. To ensure advancement and longevity of serious games in this field, we must promote standardized terminology, promote training in serious game design, and prioritize reproducibility of serious game research by creating avenues for sharing resources. This will move the field toward greater consistency, rigor, and collaboration and enable serious games to manifest their potential in neurology education more fully.
